# Gene expression analysis after receptor tyrosine kinase activation reveals new potential melanoma proteins

**DOI:** 10.1186/1471-2407-10-386

**Published:** 2010-07-21

**Authors:** Janka Teutschbein, Johannes M Haydn, Birgit Samans, Michael Krause, Martin Eilers, Manfred Schartl, Svenja Meierjohann

**Affiliations:** 1 Department of Physiological Chemistry I, Biocenter, University of Wurzburg, Wurzburg, Germany; 2 Institute of Molecular Biology and Tumor Research (IMT), University of Marburg, Marburg, Germany; 3 Department of Physiological Chemistry II, Biocenter, University of Wurzburg, Wurzburg, Germany; 4 Department of Molecular and Applied Microbiology, Leibniz-Institute for Natural Product Research and Infection Biology - Hans-Knoell-Institute, Jena, Germany; 5 Biometry and Population Genetics, Justus Liebig University Giessen, Germany

## Abstract

**Background:**

Melanoma is an aggressive tumor with increasing incidence. To develop accurate prognostic markers and targeted therapies, changes leading to malignant transformation of melanocytes need to be understood. In the *Xiphophorus *melanoma model system, a mutated version of the EGF receptor Xmrk (*Xiphophorus *melanoma receptor kinase) triggers melanomagenesis. Cellular events downstream of Xmrk, such as the activation of Akt, Ras, B-Raf or Stat5, were also shown to play a role in human melanomagenesis. This makes the elucidation of Xmrk downstream targets a useful method for identifying processes involved in melanoma formation.

**Methods:**

Here, we analyzed Xmrk-induced gene expression using a microarray approach. Several highly expressed genes were confirmed by realtime PCR, and pathways responsible for their induction were revealed using small molecule inhibitors. The expression of these genes was also monitored in human melanoma cell lines, and the target gene *FOSL1 *was knocked down by siRNA. Proliferation and migration of siRNA-treated melanoma cell lines were then investigated.

**Results:**

Genes with the strongest upregulation after receptor activation were FOS-like antigen 1 (*Fosl1*), early growth response 1 (*Egr1*), osteopontin (*Opn*), insulin-like growth factor binding protein 3 (*Igfbp3*), dual-specificity phosphatase 4 (*Dusp4*), and tumor-associated antigen L6 (*Taal6*). Interestingly, most genes were blocked in presence of a SRC kinase inhibitor. Importantly, we found that *FOSL1*, *OPN*, *IGFBP3*, *DUSP4*, and *TAAL6 *also exhibited increased expression levels in human melanoma cell lines compared to human melanocytes. Knockdown of *FOSL1 *in human melanoma cell lines reduced their proliferation and migration.

**Conclusion:**

Altogether, the data show that the receptor tyrosine kinase Xmrk is a useful tool in the identification of target genes that are commonly expressed in Xmrk-transgenic melanocytes and melanoma cell lines. The identified molecules constitute new possible molecular players in melanoma development. Specifically, a role of FOSL1 in melanomagenic processes is demonstrated. These data are the basis for future detailed analyses of the investigated target genes.

## Background

Melanoma development is a complex process based on many epigenetic and genetic factors. The most frequent genetic changes in human melanoma are activating mutations in either *BRAF *or *NRAS*. This is often combined with inactivating mutations in phosphatase and tensin homologue (*PTEN*) or cyclin-dependent kinase inhibitor 2 a (*CDKN2A*) [1].

The search for other characteristics shared between human melanoma from different individuals has revealed the importance of several proteins influencing melanoma cell cycle progression, apoptosis, cell adhesion, and angiogenesis. Examples are cyclin-dependent kinase 4 (CDK4), AKT, β-catenin, melanoma inhibitory activity protein (MIA), and Ephrin-A1 (EFNA1) [1,2]. Still, the search for further melanoma-relevant genes is a promising concept with potential therapeutic value, and several recent studies applying high-throughput gene expression profiling have associated previously unknown candidate genes with melanoma progression [3-5]. However, the comparability among different studies is low due to the variability of human tumor biopsies and the cultivation-dependent changes in melanoma-derived cell lines.

By contrast, animal models represent genetic systems with well defined genetic background where tumorigenesis is initiated by known molecular events. In the *Xiphophorus *fish melanoma model, a single oncogenic epidermal growth factor receptor, termed *Xiphophorus *melanoma receptor kinase (*xmrk*) is responsible for spontaneously developing melanoma [6].

Xmrk uses several signaling cascades that are also involved in human melanomagenesis, e.g. the phosphoinositide-3 kinase (PI3K) pathway and the RAS-RAF-MAPK cascade. Other molecules, e.g. the signal transducer and activator of transcription 5 (STAT5) and osteopontin (OPN), were first identified as essential mediators of Xmrk signaling and were subsequently shown to be relevant in human melanomagenesis as well [7-10]. These findings prompted us to search for novel Xmrk-regulated genes that may potentially play a role in human melanoma development.

It was shown many times that Xmrk signaling is highly comparable between its natural host cells - pigment cells from *Xiphophorus *- and mammalian cells that ectopically express the receptor [11-14]. Xmrk is permanently active due to its dimerization. However, to be able to differentiate between inactive and active receptor signaling, we are using the melanocytes cell line melan-a stably expressing a chimeric protein consisting of the extracellular part of EGFR ("HER") and the cytoplasmic part of Xmrk ("mrk") (melan-a "HERmrk" or shortly "Hm"). Melan-a cells lack endogenous EGFR, and the stimulation of Hm cells with EGF results in specific induction of Xmrk-dependent signaling pathways and tumorigenic transformation.

Here, we have analyzed gene expression profiles of stimulated versus unstimulated cells using a microarray approach. The genes with the strongest regulation in response to activated HERmrk were FOS-like antigen 1 (*Fosl1*), early growth response 1 (*Egr1*), osteopontin (*Opn*), insulin-like growth factor binding protein 3 (*Igfbp3*), dual-specificity phosphatase 4 (*Dusp4*), and tumor-associated antigen L6 (*Taal6*). We investigated the pathways regulating these genes and analyzed their expression in human melanoma cell lines. We furthermore found that the knockdown of *FOSL1 *reduced proliferation and migration of human melanoma cell lines. Thus, this study reveals FOSL1 as new potential molecular player in melanomagenesis by using the Xmrk melanoma model.

## Methods

### RNA isolation for microarray analysis

Cells were starved for 72 h with DMEM containing 2.5% dialyzed FCS. After stimulation with 100 ng/ml human EGF (hEGF) (tebu-bio, Le Perray en Yvelines, France) for indicated times, RNA was extracted from the cells using the RNeasy kit (Qiagen, Valencia, CA) according to the manufacturer's instructions. Only RNA samples with an A_260_/A_280 _ratio > 1.8 were used for microarray hybridization.

### Microarray probe preparation and hybridization

Transcriptional profiling was done on a microarray containing 21,168 DNA spots from the mouse cDNA library NIA 15 k and 7.4 k Mouse cDNA Clone Set (National Institute on Aging, Bethesda, MD). Total RNA was purified with RNeasy spin columns (Qiagen). After mRNA amplification with MessageAmp II aRNA Kit (Ambion, Austin, TX USA), Cy3- and Cy5-labeled cDNA probes were generated using the CyScribe cDNA Post-Labelling Kit (GE Healthcare, Amersham Place, Little Chalfont England). The labeled probes were purified with QIAquick PCR Purification Kit (Qiagen, Hilden, Germany), combined in hybridization buffer (0.2% SDS, 4.5× SSC) and hybridized on the microarray for 16 h at 55°C. Finally, the chips were washed at a stringency of 0.1 × SSC/0.1% SDS, dried by centrifugation, scanned and quantified using Scan Array Express (Perkin Elmer, Waltham, MA USA).

### Data analysis

Each experiment was performed as sandwich hybridization, i.e. instead of a coverslip, a second microarray slide was used. This provides a replicated measurement for each hybridization that can be used for quality control and that reduces technical variability. For each spot, median signal and background intensities for both channels were obtained. To account for spot differences, the background-corrected ratio of the two channels was calculated and log2 transformed. To balance the fluorescence intensities for Cy3 and Cy5 as well as to allow for comparison of expression levels across experiments, the raw data were standardized. We used the print-tip-LOWESS normalization to correct for inherent bias on each chip. Expression data and gene annotations were stored in Array Express http://www.ebi.ac.uk/arrayexpress/ (accession: E-MEXP-1311), which complies with MIAME (minimal information about a microarray experiment) guidelines. The R environment software http://www.r-project.org/ was used for data analysis. To find differently expressed genes, changes in mRNA expression levels in stimulated versus unstimulated cells were calculated for each gene. The normalized data were filtered due to strict quality criteria and analyzed using Microsoft Excel. For experimental comparisons, genes showing at least a twofold change were chosen.

### Cell culture

Mouse melanocytes transfected with HERmrk [melan-a Hm cells, [13]] or with human EGF receptor (melan-a HER) were cultured as described previously [14]. The human immortalized melanocyte cell line Hermes 3a (kindly provided by D. Bennett) was grown in RPMI supplemented with penicillin (400 U/ml), streptomycin (50 μg/ml), L-glutamine (300 μg/ml), TPA (200 nM), cholera toxin (2 pM), hSCF (10 ng/ml), endothelin (10 nM), and 10% FCS, as previously described [15]. Human melanoma cell lines Mel Im, Mel Wei, Mel Juso, and SK-Mel-3 (kind gifts by A. Bosserhoff) as well as A375, A375M, DX-3, LT5.1, and SK-Mel-28 were maintained in DMEM supplemented with penicillin (400 U/ml), streptomycin (50 μg/ml), L-glutamine (300 μg/ml) and 10% FCS. Normal human epidermal melanocytes (NHEM) derived from foreskin were obtained from PromoCell (Heidelberg, Germany) and grown in melanocyte growth medium MGM (PromoCell) under a humidified atmosphere of 5% CO_2 _at 37°C. NHEM cells were used between passages 3 and 6.

### Expression analysis by realtime PCR and pathway analysis

Cells were starved as described and subsequently stimulated with 100 ng/ml hEGF for indicated times. RNA extraction from stimulated melan-a Hm cells and human cell lines was done using Total RNA Isolation Reagent (ABgene, Epsom, UK) as recommended by the manufacturer. For the identification of pathways regulating expression of candidate genes, the small molecule inhibitors AG1478 (20 μM), PP2 (20 μM), LY294002 (10 μM), or U0126 (10 μM), respectively, were applied one hour prior to hEGF stimulation. Cells without inhibitor treatment received the equivalent amount of DMSO. Primary data of these experiments are available on request. cDNA was prepared from total RNA using the RevertAid kit with random hexamer primers (Fermentas, Burlington, Canada). PCR primers were designed using Primer3 software version 0.4.0 http://frodo.wi.mit.edu/primer3/. PCR was carried out using the iCycler IQ (Bio-Rad, Hercules, CA). Values for each gene were normalized to expression levels of β-actin (mouse cell lines) or ribosomal protein S14 (human cell lines). Primer sequences are available on request.

### Cell lysis and Western blotting

Cells were lyzed and blotted as described [16]. Before probing with the reference antibody, the blots were stripped as described. Monoclonal anti-phosphotyrosine (PY20) was from BD Biosciences (San José, CA). Phospho-p44/42 MAPK (Thr202/Tyr204) antibody was purchased from Cell Signaling Technology (Danvers, MA). Polyclonal antibodies against FOSL1 (N-17, C-12), EGR1 (C-19), OPN (P-18 and K-20), IGFBP3 (H-98), MKP2 (H-67), and ERK2 (C-14) were from Santa Cruz Biotechnology (Santa Cruz, CA). The MYC-tag antibody (9B11) was from Cell Signaling Technology (Danvers, MA). Secondary antibodies were conjugated with horseradish peroxidase and were directed against mouse (Pierce, Rockford, IL), rabbit (Bio-Rad) or goat (Abcam, Cambridge, UK). TAAL6 mouse IgM antibody (10H6) was a kind gift from S. Roffler. Secondary antibody against mouse IgM was purchased from Rockland (Gilbertsville, PA).

### siRNA transfection

One day before siRNA transfection, melanoma cells were seeded at a density of 3 × 10^4 ^cells per well of a 12-well plate. For human melanoma cells, commercially available siRNA against human *FOSL1 *as well as control siRNA (Smart Pool On Target Plus, Thermo Scientific) were used. siRNA was transfected using X-treme gene transfection reagent (Roche), according to the manufacturer's recommendations. Downregulation was monitored after 48 h by realtime PCR and Western blot analysis.

### BrdU incorporation analysis

Melanoma cell lines A375 and Mel Juso were plated in triplicate (2.5 × 10^3 ^cells per well of a 96-well plate) in DMEM containing 10% FCS. 48 h after siRNA treatment, cells were incubated with 10 μM BrdU for 6 h. BrdU incorporation was then quantified using a colorimetric BrdU cell proliferation ELISA, as recommended by the manufacturer (Roche).

### Transwell migration assay

2 × 10^4 ^A375 or Mel Juso cells were transfected with control- or *FOSL1*-specific siRNA. One day later, they were serum-starved in DMEM containing 1% dialyzed FCS for 24 h and applied to the upper chamber of a transwell inlay (polycarbonate, 10 mm diameter, 8 μm pores, Nunc). Migration was measured as described before [14]. The migration rate of the si*FOSL1*-treated cells was determined relative to the migration of control cells.

## Results

### Temporal gene expression profile after Xmrk activation

To obtain a detailed picture of the time course of Xmrk-dependent gene expression, we compared unstimulated melan-a HERmrk cells to those stimulated for 15 minutes, 1 h, 2 h, 4 h, 8 h or 24 h with EGF.

Successful receptor activation was controlled by Western blot (Additional file 1, Figure S1a) and realtime PCR (Additional file 1, Figure S1b). Both assays revealed successful stimulation, visible by receptor- and MAPK phosphorylation as well as induction of the Xmrk target gene *Opn*.

Subsequent microarray analysis revealed the regulation of 1,273 genes at one or more time-points after HERmrk stimulation. A gene was considered to be regulated when changed twofold and more. The complete list of expression data and gene annotations is available at http://www.ebi.ac.uk/arrayexpress/ (accession: E-MEXP-1311).

Regulated genes were categorized with respect to their molecular functions and biological processes according to the Gene Ontology (GO) terminology. Using the expression analysis systematic explorer (EASE) software [17], overrepresentation of gene ontology terms in the 1,273 regulated genes compared to the total number of genes assayed (21,168 spots on the chip) was calculated. Significantly enriched categories (EASE score < 0.05) are listed in Additional file 2, Table S1. Among the biological processes, protein metabolism and protein modification were particularly enriched, indicating a high metabolic activity as expected from growth factor stimulated cells, and enhancement of signal transduction processes. The most overrepresented molecular function was nucleic acid binding, encompassing transcription factors and factors regulating nucleic acid stability.

**Figure 1:**
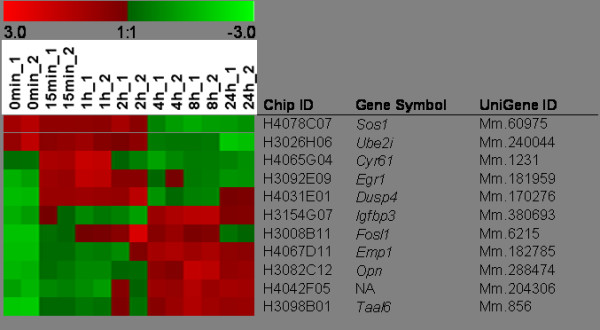
**Color map of Xmrk-specific gene expression**. Eleven highly regulated genes are itemized. RNA was extracted at time 0 (no hEGF) and at indicated time points after hEGF addition. The color code shows differences in the expression of the eleven genes regulated > 4-fold in the range of +3 (*red*) to -3 (*green*). *Sos1*, son of sevenless homolog 1; *Ube2i*, ubiquitin-conjugating enzyme E2I; *Cyr61*, cysteine rich angiogenic inducer 61; *Egr1*, early growth response 1; *Dusp4*, dual specificity phosphatase 4; *Igfbp3*, insulin-like growth factor binding protein 3; *Fosl1*, FOS-like antigen 1; *Emp1*, epithelial membrane protein 1; *Opn*, osteopontin; *Taal6*, tumor-associated antigen L6. NA: not annotated.

For further analysis, we chose eleven genes which were assigned by UniGene and which displayed more than fourfold regulation at one or more time points. *Cyr61*, *Igfbp3*, and *Opn *encode secreted proteins. SOS1 is a guanine nucleotide exchange factor. EGR1 and FOSL1 are transcription factors. EMP1 and TAAL6 are integral membrane proteins, whereas UBE2I and DUSP4 are cytosolic enzymes with ubiquitin-conjugating and phosphatase activity, respectively. Finally, the transcript with UniGene ID Mm.204306 has no assigned function. The time-dependent course of gene expression is depicted in a color map (Figure 1). Genes regulated at early time points were for example *Cyr61 *and *Egr1*, while *Emp1 *and *Taal6 *were regulated at late stimulation times.

**Figure 2:**
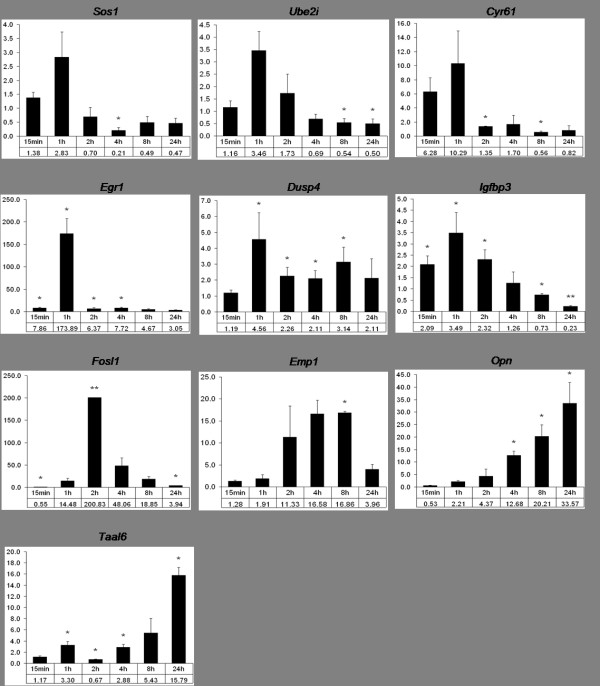
**Validation of microarray results of ten candidate genes by quantitative realtime PCR**. RNA was extracted at indicated time points after HERmrk stimulation with hEGF. Expression of candidate genes was compared to unstimulated cells. The fold change of transcript, referred to the unstimulated control, which is set as 1, is indicated on the y axis. Mouse β-actin served as reference gene. Significant regulation (p < 0.05) is marked by an asterisk; two asterisks indicate highly significant regulation (p < 0.001) (Student's t-test, paired, one-tailed).

To validate the microarray results for highly regulated genes, we used quantitative realtime PCR (Figure 2). The time course already observed in the microarray experiment was largely confirmed by realtime PCR analysis. *Sos1*, *Ube2i*, *Cyr61 *and *Egr1 *were mainly upregulated after short stimulation times and decreased later. The expression of *Dusp4 *was highest after 1 h, but an upregulation in comparison to the unstimulated control was visible until 24 h. In case of *Igfbp3*, the situation looked slightly different compared to the microarray experiment. While in the latter the gene was found to be upregulated after 15 min and again from 4 to 24 h, the transcription induction was only visible at early times when analyzed by realtime PCR. For *Fosl1*, the expression was highest after 2 h (200-fold) and decreased later, similar to the situation observed in the microarray experiment. Expression levels of *Emp1 *were strongly increasing from 2 to 8 h and decreased to 4-fold at 24 h. The late-responding genes *Opn*, *Taal6*, and the unnamed gene product (UniGene ID Mm.204306, data not shown) steadily increased with the highest levels at 24 h. However, as the gene with UniGene ID Mm.204306 was retired from UniGene during the course of our experiments, we omitted it from further analyses.

Altogether, in all cases except *Igfbp3*, transcript induction as well as its time course was confirmed by realtime PCR.

### Identification of signaling pathways

Previous analyses have revealed that Xmrk transfers signals via STAT5, the PI3K pathway, the RAS-RAF-MAPK cascade, and the cytoplasmic SRC kinase family member FYN [6]. To identify the pathways responsible for modulation of the chosen ten genes, we blocked known Xmrk-induced pathways using the small molecule inhibitors AG1478, U0126, PP2, or LY294002. Target gene expression in presence or absence of the inhibitors was analyzed by realtime PCR (Additional file 3, Table S2 and, for a schematic overview, Addditional file 4, Figure S2).

As expected, the inducing effects of EGF on all genes were abrogated when HERmrk was inhibited by AG1478. While regulation of *Emp1*, *Fosl1*, and *Opn *was both MEK- and SRC family kinase-dependent, induction of *Sos1*, *Ube2I*, *Dusp4*, and *Taal6 *was only restrained by inhibiting SRC-family kinases with PP2. *Egr1 *expression could only be decreased after MEK inhibition, and *Cyr61 *transcription was dependent on MEK and PI3K. Finally, expression of *Igfbp3 *was decreased after application of each of the inhibitors.

### Expression of candidate genes in human melanoma cell lines

For further analysis, we focused on six significantly regulated genes from four different functional groups: the transcription factors *FOSL1 *and *EGR1*, the secreted proteins *OPN *and *IGFBP3*, the phosphatase *DUSP4*, and the membrane protein *TAAL6*. We monitored their expression levels in human melanoma cell lines compared to normal human epidermal melanocytes (NHEM). For this analysis we chose eight different cell lines, containing either activating N-RAS- (DX-3, LT5.1 and Mel Juso) or B-RAF mutations (A375, A375M, Mel Wei, Mel Im, SK-Mel-3 and SK-Mel-28).

**Figure 3:**
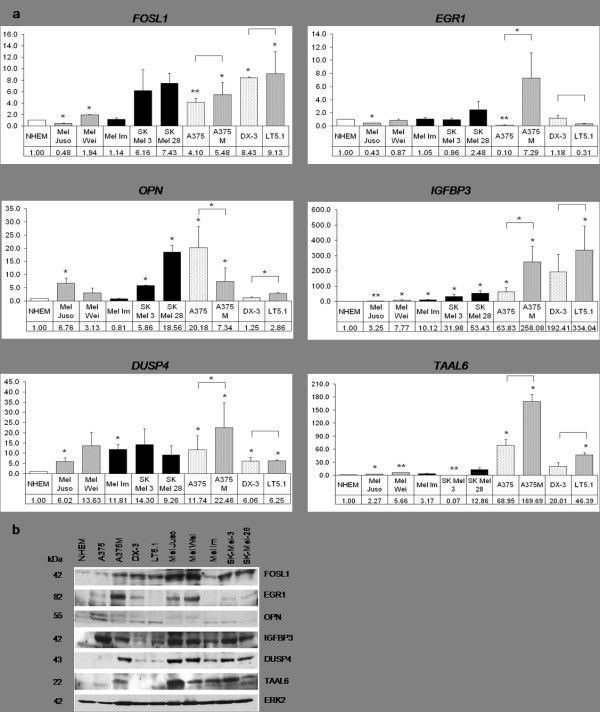
**Expression of candidate genes in human melanoma cell lines**. *a*, Realtime PCR was performed to determine *FOSL1*, *EGR1*, *OPN*, *IGFBP3*, *DUSP4 *and *TAAL6 *expression in human melanoma cell lines compared to NHEM cells. Fold change of transcript, referred to NHEM cells, is indicated. β-actin was used as reference gene. Significant (p < 0.05) or highly significant (p < 0.001) regulation are marked by one or two asterisks, respectively (Student's t-test, paired, one-tailed). *b*, Western blot analysis of the candidate genes in human melanoma cell lines. Expression of ERK2 served as loading control. Please note that the order of cell lines differs from *A*. On the OPN blot, the lower band represents the OPN protein. For TAAL6, the upper band corresponds to the protein of the expected size.

Realtime PCR revealed a significantly higher expression of *FOSL1*, *OPN*, *IGFBP3*, *DUSP4 *and *TAAL6 *in most of the melanoma cell lines compared to normal melanocytes (Figure 3a). The sole exception was observed in case of *EGR1*, where only A375M cells displayed a significant upregulation. At the level of protein, NHEM displayed almost no expression of most of the proteins, whereas in the majority of examined melanoma cell lines FOSL1, IGFBP3 and DUSP4 were strongly expressed (Figure 3b). EGR1, OPN and TAAL6 were expressed in at least two third of the cell lines, but not in NHEM cells.

**Figure 4:**
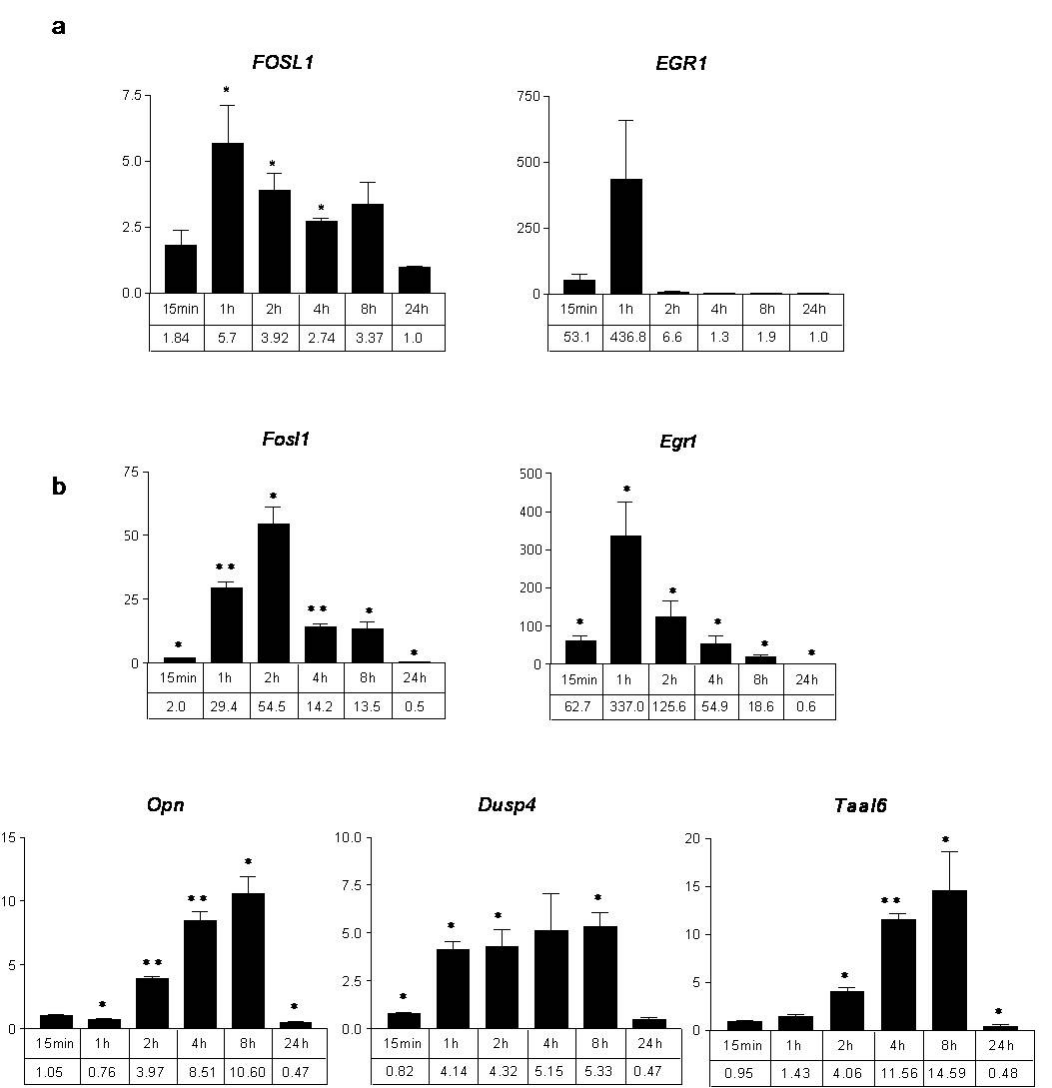
**Expression of candidate genes in response to stimulation of human EGFR**. Realtime PCR was performed to determine expression levels of *FOSL1 *and *EGR1 *in the human melanoma cell line A375 (*a*) or expression levels of *Fosl1*, *Egr1*, *Opn*, *Dusp4*, and *Taal6 *in *HER*-transgenic melan-a cells (*b*), stimulated with EGF for the indicated times. The fold change of transcript, referred to the unstimulated controls, is indicated on the y axis. Murine and human β-actin were used as reference genes. Significant (p < 0.05) or highly significant (p < 0.001) regulation are marked by one or two asterisks, respectively (Student's t-test, paired, two-tailed).

As Xmrk is an orthologue of the human EGF receptor, we wanted to elucidate if the upregulation of the identified target genes is a shared property between human EGFR and Xmrk/HERmrk. Of the 10 C-terminal SH2 docking sites of human EGFR, most of them with overlapping substrate specifity, 7 are conserved in Xmrk, suggesting at least partial functional similarity. The melanoma cell line A375 reportedly expresses human EGFR and responds to addition of EGF [18]. When induction of *FOSL1*, *EGR1*, *OPN*, *IGFBP3*, *DUSP4 *and *TAAL6 *was monitored after EGF stimulation between 15 minutes and 24 h, only the fast responding *FOSL1 *and *EGR1 *genes were found to be induced (Figure 4a and data not shown). Compared to HERmrk-expressing melanocytes, *FOSL1 *upregulation was weaker in A375, while *EGR1 *induction was even stronger (compare Figure 4a and Figure 2). As A375 cells express oncogenic BRAF^V600E ^and already underwent the process of transformation, it is possible that ongoing endogenous aberrant signaling concealed EGFR stimulation in this cell line. For this reason, and to gain a better comparison to the untransformed melan-a HERmrk cells, we used melan-a cells stably transfected with human EGFR ("melan-a HER") and performed an experiment similar to the one performed with A375 cells (Figure 4b). Here, all investigated genes except *Igfbp3 *were upregulated in response to EGF. Apart from the downregulated *Opn *and *Taal6 *values at 24 h, the extent and time course of stimulation were comparable between HERmrk and HER stimulation (Figures 2 and 4b).

**Figure 5:**
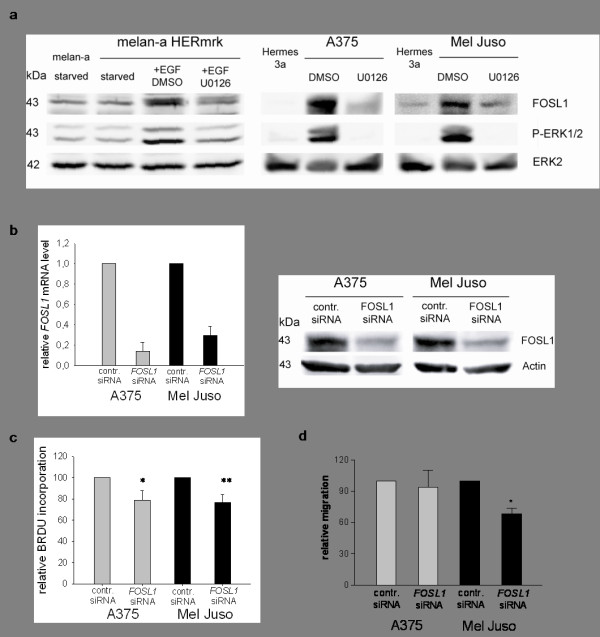
**MAPK-pathway dependent expression of *FOSL1 *and effects of *FOSL1 *knockdown on proliferation and migration of melanoma cells A375 and Mel Juso**. *a*, Western blot analysis of FOSL1 levels in murine melanocytes (left) and human melanocytes and melanoma cell lines (right). Murine melan-a and melan-a HERmrk cells were starved for 2 days before EGF and U0126 were added for 24 hours. Human melanoma cells were treated with U0126 for 24 hours. P-ERK1/2 levels were used to verify MEK inhibition, ERK2 was used as reference. *b*, Realtime PCR analysis (left) and Western blot analysis (right) of *FOSL1 *expression levels after treatment of the indicated cell lines with control siRNA or *FOSL1*-specific siRNA. For realtime PCR analysis, control siRNA-treated *FOSL1 *levels were set as 1. For the Western blot, β-actin was used as reference. *c*, BrdU incorporation of A375 and Mel Juso melanoma cells transfected with control siRNA or *FOSL1*-specific siRNA. *d*, Migration of A375 and Mel Juso cells treated with *FOSL1*-specific siRNA relative to control siRNA-treated cells. Significant (p < 0.05) or highly significant (p < 0.001) differences between control and *FOSL1 *siRNA treated cells are marked by one or two asterisks, respectively (Student's t-test, paired, two-tailed).

Among the genes identified, the protein encoded by *FOSL1 *constitutes an interesting candidate with a potential effect on melanoma biology. It is part of the AP-1 complex, which is a functional downstream target of the MAP kinase pathway that is commonly activated in melanoma [19]. Furthermore, c-JUN, which might be a potential binding partner for FOSL1 in the AP-1 complex, is highly expressed in most melanoma and is required for tumor transformation [20-22]. The human protein atlas database constitutes a platform which offers an extensive amount of protein expression data gained from a large variety of normal human tissues, cancer tissues and cell lines [23,24]. Here, FOSL1 expression is low or non-detectable in most tissues, and moderate in epidermal skin cells. Among melanoma tissues, two thirds express moderate or high levels of the protein, and both melanoma cell lines investigated also show high expression http://www.proteinatlas.org/tissue_profile.php?antibody_id=4396&g_no=ENSG00000175592. These data confirm our own observations, namely the increase of FOSL1 expression in transformed or activated pigment cells. In our study, FOSL1 protein levels were not only upregulated in mouse melanocytes expressing HERmrk, but were also elevated in human melanoma cell lines compared to the human melanocyte cell line Hermes3a (Figure 5a) and NHEM cells (Figure 3b). Inhibition of MEK strongly reduced FOSL1 protein in HERmrk-transgenic cells as well as in the human cell lines A375 and Mel Juso (Figure 5a). This suggests that MAPK pathway activation by BRAF^V600E ^(as in A375) and by NRAS^Q61K ^(as in Mel Juso) is important in maintaining FOSL1 expression. To investigate the effect of FOSL1 on melanoma growth, we downregulated *FOSL1 *in the melanoma cell lines A375 and Mel Juso using siRNA (Figure 5b). Proliferation was monitored by BrdU incorporation assay, which indicates the number of cells in S phase (Figure 5c). In both cell lines, BrdU incorporation was significantly reduced in presence of *FOSL1 *siRNA (79 and 77%, respectively). Furthermore, the migration capacity of Mel Juso cells was reduced to 66% (Figure 5d).

## Discussion

Here we describe the regulation of potential novel melanoma candidate genes using an inducible version of the EGFR orthologue Xmrk.

Ten of the most strongly regulated genes were examined in presence of pathway inhibitors to determine the respective signal transduction chain responsible for their regulation. Eight out of ten genes were at least partly controlled by members of the SRC kinase family, while six genes were induced by the MAPK pathway and only two genes by the PI3 kinase pathway. Usually, gene regulation was reduced by inhibition of more than one pathway, which indicates an interplay between the simultaneously activated pathways. Although we could only perform the inhibitor analysis with a small group of genes, which is not representative of the overall mode of gene regulation, it was surprising that such a high gene number was regulated by SRC kinases. From previous studies it is known that FYN is the only SRC family kinase member that is directly activated by Xmrk [25]. FYN prevents inactivation of MAPK by reducing MAPK phosphatase 1 (MKP-1) expression, but also stabilizes the complex between Xmrk and the p85 subunit of PI3K [13,26]. This might explain why in our study many genes are not only affected by SRC family kinase inhibition, but also by blocking MEK or PI3K. Although the effect of SRC kinases on gene expression has not yet been investigated in human melanoma, accumulating data indicate that this pathway plays a vital role for the malignant cells. Specifically, it was shown by kinase activity profiling that SRC is activated in primary human melanoma and its inhibition leads to reduced growth [27]. Activated SRC was also detected in cutaneous, mucosal and metastatic melanoma, and its inhibition by dasatinib or bosutinib blocked the growth of several melanoma cell lines [28,29]. Furthermore, an active SRC family kinase-focal adhesion kinase complex, responsible for migration and metastatic spread, was described both for the Xmrk model and for human melanoma [14,30]. Together with our own data, this depicts an important melanomagenic role for SRC kinases in general and raises the necessity to further scrutinize SRC family kinase-dependent gene regulation in human melanoma.

Among the identified genes, expression of *Egr1, Dusp4, Igfbp3, Fosl1, Opn*, and *Taal6 *were investigated more closely. Importantly, five of these genes have been found to be upregulated in several different melanoma microarray studies. Recently, B-RAF^V600E^-dependent genes were analysed by MEK inhibition or *BRAF *siRNA in human melanoma cell lines, revealing regulation of the transcription factors *FOSL1 *and *EGR1 *by this pathway [31]. Interestingly, EGR1 belongs to a distinct group of salivary marker genes expressed in melanoma-bearing mice [32]. *IGFBP3 *was more strongly expressed in metastatic and cutaneous melanoma compared to melanocytes [33]. Its function seems to depend on the cellular context, as both anti- and pro-tumorigenic roles were attributed to this protein [34,35].

*DUSP4 *and *OPN *were upregulated in cutaneous melanoma in comparison to normal skin or benign nevi [5]. DUSP4 is a dual phosphatase capable of dephosphorylating p38, JNK1 and ERK1/2, though JNK1 seems to be the preferred target in vitro [36]. Its upregulation may simply reflect counterregulation of the ERK1/2 pathway in melanoma, but has not yet been investigated more closely. OPN, on the other hand, is a well-known melanoma marker [37,38] and served as internal control in our studies.

Finally, in a murine melanoma model with xenografted A375 cells, *EGR1 *and *OPN *expression was enhanced in cells derived from metastatic melanoma compared to the parental cell line as well as in metastatic melanoma versus primary melanoma [39]. The original data of all above-mentioned microarray studies are available at http://www.oncomine.org.

Due to the important role of the AP-1 complex in human melanoma, the lack of information on FOSL1 function in this tumor type, and the MAPK pathway dependent induction of *FOSL1 *in melanoma cell lines, we investigated the effect of *FOSL1 *knockdown on the proliferation of two melanoma cell lines and found a significant inhibition of cells entering the S phase, which was not due to the induction of apoptosis (data not shown). Also, migration of Mel Juso cells was decreased after *FOSL1 *knockdown. Its function in melanoma was not described previously, but pro-tumorigenic roles of FOSL1 were reported for other solid cancers [40,41]. Oncogenic EGFRvIII in glioblastoma cells induces *FOSL1 *[41], and it modulates the malignant features of glioma cells, so it was suggested as target for therapeutic interventions against malignant gliomas [42]. An oral DNA vaccine directed against FOSL1 has been demonstrated to effectively suppress tumor growth, angiogenesis and metastasis in mice injected with breast carcinoma cells [43].

In summary, we used the high overlap between pathways downstream of Xmrk and established human melanoma pathways for the search of new melanoma-relevant target genes. Our gene and protein expression results indicate that Xmrk serves as a suitable model oncogene for this purpose. As a proof of principle, we investigated the AP-1 complex component FOSL1 in more detail. We found that the gene is similarly regulated in a MAPK-dependent manner by Xmrk and by human melanoma oncogenes. Importantly, we also could demonstrate a pro-tumorigenic role of *FOSL1 *in human melanoma cell lines, thus confirming the Xmrk oncogene as instrumental in the search of new melanoma players.

## Conclusions

Here we show the receptor tyrosine kinase Xmrk is a valuable tool to identify target genes and proteins that are commonly shared between Xmrk-transgenic melanocytes and human melanoma cell lines. Interestingly, of the ten most strongly upregulated genes, the majority was regulated by SRC kinases, followed by the MAPK pathway. The transcription factor component gene *FOSL1 *also belongs to this group of genes. By knocking down *FOSL1*, we could demonstrate a pro-proliferative and pro-migratory function of this protein in melanoma cell lines. The presented data reveal new potential melanoma-relevant genes that can now be investigated for their melanomagenic function in detail.

## List of abbreviations used

AKT: v-akt murine thymoma viral oncogene homolog; AP-1: activator protein 1; BRAF: v-raf murine sarcoma viral oncogene homolog B1; CDKN2A: cyclin dependent kinase inhibitor 2A; CYR61: cysteine-rich, angiogenic inducer 61; DUSP4: dual specificity phosphatase 4; EGF: epidermal growth factor; EGFR: epidermal growth factor receptor; EGR1: early growth response 1; EMP1: epithelial membrane protein 1; ERK: extracellular regulated MAP kinase; FOSL1: FOS-like antigen 1; FYN: FYN oncogene related to SRC, FGR, YES; HER: human epidermal growth factor receptor; HERmrk: chimera of HER and Xmrk; hSCF: human stem cell factor; IGFBP3: insulin-like growth factor binding protein 3; JNK1: JUN N-terminal Kinase 1; c-JUN: c-JUN oncogene; MAPK: mitogen-activated protein kinase; MKP-1: MAK kinase phosphatase 1; NHEM: normal human epidermal melanocyres; NRAS: neuroblastoma RAS viral (v-ras) oncogene homolog; OPN: osteopontin; PI3K: phosphoinositide-3-kinase; PTEN: phosphatase and tensin homolog; siRNA: small inhibitory RNA; SOS1: son of sevenless homolog 1; SRC: v-src sarcoma (Schmidt-Ruppin A-2) viral oncogene homolog; STAT5: signal transducer and activator of transcription 5; TAAL6: tumor-associated antigen L6; TPA: 12-O-Tetradecanoylphorbol-13-acetate; UBE2I: ubiquitin-conjugating enzyme E2I; Xmrk: Xiphophorus melanoma receptor kinase

## Competing interests

The authors declare that they have no competing interests.

## Authors' contributions

SM and MS conceived and designed the experiments. JMH and JT performed all cell culture based analyses. BS and MK performed microarray analysis and data analysis. SM and JT wrote the manuscript. JMH, ME, and MS helped writing the manuscript and contributed to discussions. All authors read and approved the final manuscript.

## Pre-publication history

The pre-publication history for this paper can be accessed here:

http://www.biomedcentral.com/1471-2407/10/386/prepub

## Supplementary Material

Additional File 1 **Figure S1 Activation of the chimeric receptor HERmrk in melan-a cells**. *a*, Stimulation of HERmrk with hEGF for indicated time periods resulted in autophosphorylation of the receptor (*top*) and phosphorylation of the downstream factor MAPK (*bottom*). *b*, Gene expression of the known Xmrk target *Opn *was induced after activation of HERmrk with hEGF. The fold change of transcript, referred to the unstimulated control, which is set as 1, is indicated on the y axis. Murine β-actin served as reference gene. Click here for file

Additional file 2 **Table S1 Expression analysis systematic explorer (EASE) report of differentially expressed genes**. EASE analysis was performed with genes that were regulated > 2-fold in microarray analysis. Only selected categories with an EASE score < 0.05, i.e. categories with enrichment of differentially expressed genes, are listed. The number of regulated genes belonging to the category is quoted in the column *List Hits*. Click here for file

Additional file 3 **Table S2 Pathways for regulation of genes by activated HERmrk**. Time and manner (up or down) of maximal regulation are itemized in the third column. Small molecule inhibitors AG1478, U0126, PP2, or LY294002 were applied to inhibit HERmrk, the MAPK kinase MEK, SRC-family kinases, or PI3K, respectively. Effects of inhibitors on expression of the candidate genes at indicated time points were monitored by realtime PCR. "+" indicates inhibition of Xmrk-dependent gene expression changes, "-" symbolizes that there was no effect. Click here for file

Additional file 4 **Figure S2 Schematic overview of the pathways induced by HERmrk and the subsequent induction of indicated genes, as shown in this manuscript.**. Genes marked with an asterisk were only induced by one of the investigated pathways, while the induction of genes without asterisk was effected by three (*Igfbp3*) or two pathways (all other genes). The inhibitors used in this manuscript are depicted in red. AG1478 inhibits EGFR and its orthologues, including Xmrk. U0126 blocks MEK, LY294002 inhibits PI3 kinase, and PP2 inhibits SRC family kinases (FYN being the only one activated by Xmrk). Click here for file
