# Supplementary figures and images for: Gene expression analysis after receptor tyrosine kinase activation reveals new potential melanoma proteins
Source: BMC Cancer. 2010 Jul 21;10:386. doi: 10.1186/1471-2407-10-386 (PMC2912872; doi:10.1186/1471-2407-10-386)

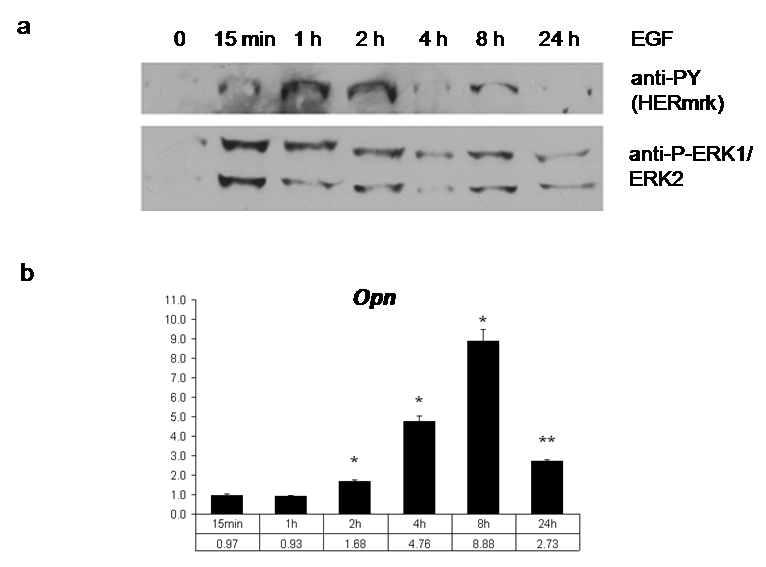

Supplement: Additional File 1 — Figure S1 Activation of the chimeric receptor HERmrk in melan-a cells. a, Stimulation of HERmrk with hEGF for indicated time periods resulted in autophosphorylation of the receptor (top) and phosphorylation of the downstream factor MAPK (bottom). b, Gene expression of the known Xmrk target Opn was induced after activation of HERmrk with hEGF. The fold change of transcript, referred to the unstimulated control, which is set as 1, is indicated on the y axis. Murine β-actin served as reference gene. [file 1471-2407-10-386-S1.PNG]

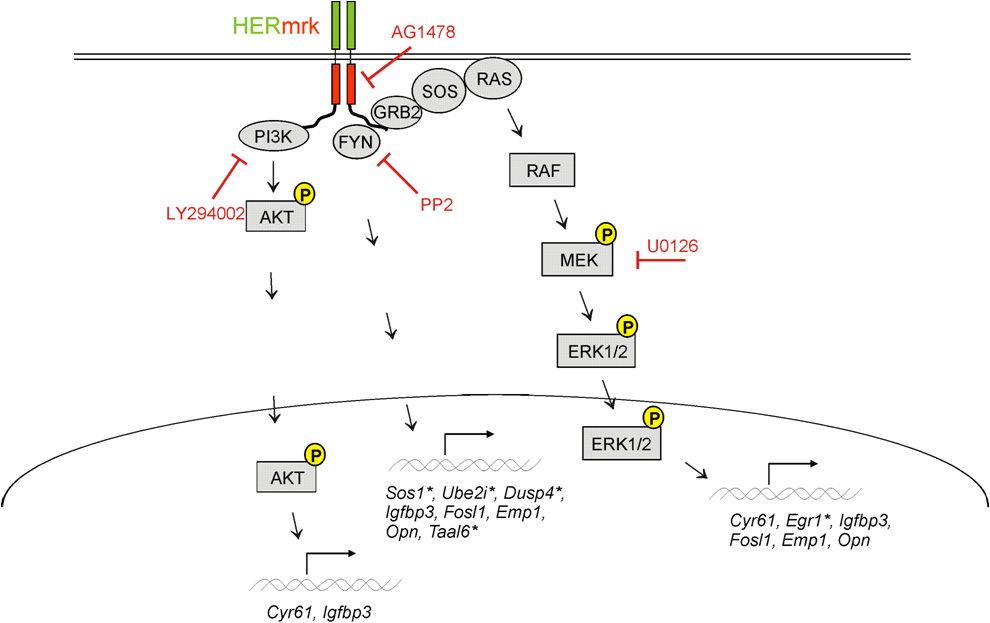

Supplement: Additional file 4 — Figure S2 Schematic overview of the pathways induced by HERmrk and the subsequent induction of indicated genes, as shown in this manuscript.. Genes marked with an asterisk were only induced by one of the investigated pathways, while the induction of genes without asterisk was effected by three (Igfbp3) or two pathways (all other genes). The inhibitors used in this manuscript are depicted in red. AG1478 inhibits EGFR and its orthologues, including Xmrk. U0126 blocks MEK, LY294002 inhibits PI3 kinase, and PP2 inhibits SRC family kinases (FYN being the only one activated by Xmrk). [file 1471-2407-10-386-S4.PNG]
